# Political bias indicators and perceptions of news

**DOI:** 10.3389/fpsyg.2023.1078966

**Published:** 2023-04-26

**Authors:** Kathryn Bruchmann, Subramaniam Vincent, Alexandra Folks

**Affiliations:** ^1^Department of Psychology, Santa Clara University, Santa Clara, CA, United States; ^2^Markkula Center for Applied Ethics, Santa Clara University, Santa Clara, CA, United States

**Keywords:** bias indicator, news media, media literacy, political bias, news bias

## Abstract

**Introduction:**

Recently, a variety of political bias indicators for social and news media have come to market to alert news consumers to the credibility and political bias of their sources. However, the effects of political bias indicators on how people consume news is unknown. Creators of bias indicators assume people will use the apps and extensions to become less biased news-consumers; however, it is also possible that people would use bias indicators to confirm their previous worldview and become more biased in their perceptions of news.

**Methods:**

Across two studies, we tested how political bias indicators influence perceptions of news articles without partisan bias (Study 1, *N* = 394) and articles with partisan bias (Study 2, *N* = 616). Participants read news articles with or without political bias indicators present and rated the articles on their perceived political bias and credibility.

**Results:**

Overall, we found no consistent evidence that bias indicators influence perceptions of credibility or bias in news. However, in Study 2, there was some evidence that participants planned to use bias indicators in the future to become more biased in their future news article selection.

**Discussion:**

These data shed light on the (in) effectiveness of interventions against blindly consuming biased news and media.

## Introduction

With the onset of the “Fake News” era and extreme political polarization in the USA, several tools have been developed to spot and mitigate the effects of news bias, such as fact checking websites, apps, and political bias indicators. Political bias indicators tag news articles and social media posts with their political leaning and/or their factual accuracy by using machine learning algorithms to analyze words and images for the purpose of identifying partisan language (Yu et al., 2008). Sites like *allsides.com* (Allsides, 2021) and *mediabiasfactcheck.com* (Media Bias/Fact Check, 2021) seek to uncover bias for thousands of articles and sources by providing services where users actively search out information about news sources including political bias while they are reading news. More contemporary are Google Chrome extensions like *Nobias.com* or *TheFactual.com* that provide visual labels of article (or source) bias on search results or social media shares before a user has even opened the article. But, there is little information available about how news-consumers actually *use* these bias indicators. While some may argue that there are clear benefits of transparency with the presence of bias indicators, others question how political bias indicators actually influence the way people read and perceive news articles. The purpose of the present paper is to test *if* and *how* the presence of political bias indicators changes readers’ perceptions of news, and to understand how people perceive bias indicators themselves.

Organizations that create bias indicators seem to operate under the assumption that bias indicators help readers correct their own biases, or to become more aware of the political leaning of news media. In effect, they seem to believe that bias indicators might help readers have greater news media literacy; specifically in that they would develop awareness of the political lean of news even if from unfamiliar sources. However, given people’s tendency to seek out and agree with pro-attitudinal and worldview confirming information (e.g., Knobloch-Westerwick and Meng, 2009), it is also possible that bias indicators make people more biased in their perceptions and selection of news media. The present research provided the first exploratory test of whether the presence of bias indicators helps people become more news media literate, or more world-view confirming (or neither) in their perceptions of news.

## Bias indicators as news literacy cues

In general, news media literacy is touted as a promising antidote for the spread of misinformation (e.g., Bulger and Davison, 2018; for a review of other strategies to correct misinformation, see Lewandowsky et al., 2012) in part because media literacy has been shown to increase skepticism toward news (Vraga and Tully, 2018). However, developing news media literacy requires effort; specifically, people who are more news literate have knowledge about the content in the news, but also understand how things like news production or personal beliefs can influence the way news is interpreted (Craft et al., 2016). People who have more news literacy also perceive that they are more in control over the influence that media might have on them (Maksl et al., 2013). Presumably, the visual tags of a bias indicator could provide a heuristic cue for readers to help increase their news literacy with little to no effort on their part. As a matter of fact, Nobias describes the need for their product by saying “to make successful decisions [about how valuable information is] one needs to be more skeptical, more vigilant, more rigorous, and invest more labor and time than ever before” (Nobias, 2021).

There is some evidence that this type of news media literacy cue can indeed help people become less susceptible to misinformation. For example, explicitly warning people that they are about to encounter false or biased information can be useful in protecting against persuasion (i.e., “pre-bunking”; Chambers and Zaragoza, 2001; Compton, 2013; van der Linden et al., 2020; Lewandowsky and van der Linden, 2021), but there is evidence that these warnings need to be given repeatedly and be specific about which information specifically is inaccurate (Marsh and Fazio, 2006). One experiment testing perceptions of news articles shared on Facebook found that articles tagged with labels of “disputed” or “rated false” were rated as less accurate than a control (Clayton et al., 2019; see also Bode and Vraga, 2015 col), demonstrating that these kinds of tags can increase scrutiny or skepticism the same way that media literacy might. And, research examining the effectiveness of an intervention created by Facebook giving readers “tips to spot false news” also led to a decrease in perceived accuracy of fake news articles (Guess et al., 2020); regardless of the political leaning of the articles.

## Bias indicators as worldview confirming

However, another way that bias indicators might influence people’s perceptions of news is by indicating whether a news article will be worldview confirming– by signaling political beliefs– or not. A myriad of research has examined people’s tendency to seek out pro-attitudinal or worldview-confirming information (for a review, see Knobloch-Westerwick, 2015), particularly in the domain of politics (e.g., Iyengar et al., 2012). One explanation for this type of confirmation bias is people’s political social identity; decades of research suggest that people like and trust political ingroup members more than outgroup members (e.g., Tajfel and Turner, 1979; Byrne, 1997), in part because they view their political ingroup members as sharing moral values (Bruchmann et al., 2018). Other research suggests that it is actually a dislike for the political outgroup more than a like for the political ingroup that could drive these effects (i.e., negative partisanship, e.g., Abramowitz and Webster, 2018).

As such, if people learn that a news article is aligned with their political beliefs it might lead them to like and trust it more, or perhaps finding a news article to be counter to their political beliefs might lead people to like and trust it less. Indeed, research (Slothuus and de Vreese, 2010) has demonstrated that people are more persuaded by political arguments that are ostensibly from political ingroups than outgroups, suggesting that articles tagged in politically consistent ways would be viewed as more credible or persuasive, and corrections to misinformation are more successful at changing opinions when they do not threaten people’s worldview [see Jerit and Zhao (2020) for a review]. Thus, if a bias indicator makes a newsource seem like it will be worldview confirming, it may be viewed as more credible or trustworthy, and people might rate stories marked as biased in their own political direction to be viewed as more credible.

### Political ideology^1^

People’s own political ideology might influence perceptions of news in other ways, as well. While there is some evidence that political ingroup bias is equally likely for liberals and conservatives (Chambers et al., 2013), and liberals and conservatives are equally likely to try to avoid information from the other side (e.g., Frimer et al., 2017), other research suggests that conservatives are more likely to reject counter-attitudinal information than liberals (Fessler et al., 2017).

Additionally, trust in news has decreased for U.S. Americans (Pew Research Center, 2022)– something that many bias-indicator sites are attempting to address– and there is consistent research demonstrating that political conservatives feel much more negatively about news media in general (Pew Research Center, 2020), and view news media to be liberally biased across the board (Mitchell et al., 2020). In fact, research from the Knight Foundation (2020) suggests that almost 70% of Republicans have an unfavorable view of the media, versus only 20% of Democrats.

As such, political bias indicators might influence political liberals versus conservatives differently. It is possible that conservatives might be more influenced by bias indicators, in that they might provide a greater signal of credibility or legitimacy to news; or that they might provide more (less) confidence in worldview-(dis)confirming information. However, because of the mixed findings of previous research, these questions are exploratory.

## The present research

The goals of the present research are four-fold. First, we aim to test whether and how political bias indicators influence the way U.S. Americans read and perceive news, and whether partisanship plays a role in this influence. And second, this research will examine how people perceive bias indicators themselves and how people intend to use them as part of their news consumption. Specifically, we asked the following four research questions.

RQ1: Do bias indicators influence how politically biased people perceive news articles to be?

RQ2: Do bias indicators influence how credible people perceive news articles to be?

RQ3: Do people believe that bias indicators are accurate representations of article bias?

RQ4: How do people predict they would use bias indicators in the future?

Across two exploratory experiments, participants who identified as either liberal or conservative read news stories that included bias indicators or not. In Study 1, participants read a neutral news story (as determined by NoBias), and in some cases received a bias indicator label that said it was right-leaning, left-leaning, or center-leaning. After reading, participants rated the credibility and perceived bias of the article. In Study 2, participants read an article that was actually biased either liberally or conservatively (as determined by NoBias), and saw a bias indicator that either indicated the bias (in the direction determined by NoBias), or falsely stated that the article was unbiased. Participants then rated the credibility and political bias of the articles (Studies 1 and 2), their perceptions of the accuracy of bias indicators (Studies 1 and 2), and their intentions of how to use bias indicators in the future (Study 2).

These two experiments allowed for a test between the two competing hypotheses: bias indicators as shortcuts for media literacy, or bias indicators as world-view confirming tools. If bias indicators increase media literacy and add an overarching cue of (il)legitimacy to the articles, we would predict that articles labeled as politically biased (in either direction) would be perceived as less credible than articles labeled as having no bias, or articles without a label. However, if bias indicators are instead used in a worldview confirming way, we predict that partisans would view articles that were biased in the same (opposing) political direction and would view them as more (less) credible.

## Study 1

### Materials and methods

#### Participants and design

U.S. American MTurk workers (*N* = 394) were compensated $2.50 to complete a study called “Perceptions of Journalism.” Participants ranged in ages from 19 to 74 (*M*_age_ = 36.8 years, *SD*_age_ = 16.4 years), and a majority were men (65.2%) and white (76.9%; 9.87% were Black or African-American, 9.64% were Hispanic or Latino, 6.28% were Asian, and less than 2% were other races). Participants’ areas of residence largely followed typical demographic trends in the USA with most participants living in the South (32.2%), the West (30%) followed by the Northeast (22.6%), and the Midwest (22.3%).

Consistent with the US nationwide demographics (Pew Research Center, 2020), more participants identified as Democrat (42.6%) than Republican (33.2%); however, a smaller portion of our sample identified as Independent or Other (25.2%) than in the general population. Because we were interested in whether the bias indicators matched the political ideology of the participants or not, the sample was bifurcated to be liberal (*N* = 193) or conservative (*N* = 159). Participants who did not lean right or left (*N* = 42) were excluded from analyses, leaving a final sample of *N* = 352.

Both studies were approved by the Santa Clara University human subjects committee (approval number: 19-04-1260). Upon starting the study, participants provided consent, and were randomly assigned to view an article with one of four bias indicators: left-leaning bias, right-leaning bias, center bias, or the no-indicator control.

#### Materials

##### Articles

The research team chose three articles covering topics without a current partisan angle to them that were between 600 and 900 words. The three articles were determined to be highly credible and “center-leaning” by the NoBias Google Chrome extension. The Nobias software uses machine learning technology to verify patterns of word usage that match significant phrases and text patterns with those used by liberals and conservatives in order to establish an ideological “slant index” (Gentzkow and Shapiro, 2010). The first article was “Did John Wilkes Booth get away with murdering President Abraham Lincoln?” from *The Philadelphia Inquirer* (Colimore, 2019). The other two articles were “South Florida, in effort to save tourism industry, may spend millions to remove seaweed invading beaches” from *Fox News* and “Rates are low, and mortgages are cheap. So why aren’t Americans buying more homes?” from *CNN* (Shirazi, 2019; Tappe, 2019). In order to control for perceptions of source familiarity, credibility, or bias, the research team re-formatted all articles to appear to be shared by the *Quad City Times*, a center-leaning (per analysis of *MediaBias/FactCheck*, The Factual, and NoBias) and small regional news source in the upper Midwestern United States.

##### Bias indicators

**FIGURE 1:**
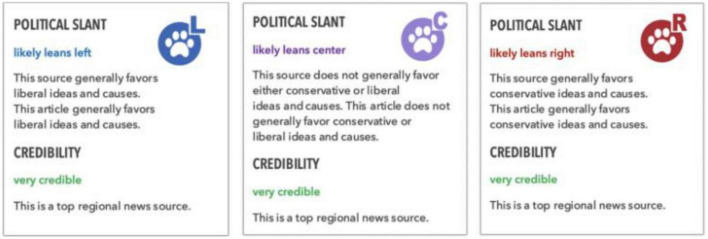
Sample bias indicators used in Study 1 and 2. Reproduced with the permission of NoBias, LLC.

Each article (aside from the control group) included a bias indicator label. These were created by using images of bias indicators from *NoBias.com*. The image included a paw print colored with either red (leans right), blue (leans left) or purple (leans center), and written information about the political slant of the article (leans left, leans right, leans center) and of the source (labeled as ‘‘very credible’’ for all conditions).^2^ The bias indicators were displayed three times on each article: at the top, in the middle, and at the bottom of the article. See Figure 1 for example bias indicators. The control condition did not contain any bias indicators.

#### Procedure

First, participants read instructions asking them to examine how “political bias indicators” on websites can influence perceptions of news articles. They saw sample bias indicators in order to learn what the symbols meant, and learned they would be reading articles and answering questions about what they read. Then, participants read one of the articles with (or without, for control) the No Bias indicator images.

##### Article questions

In line with the cover story, after finishing the news article, participants answered multiple choice questions that tested their comprehension or memory of the article (e.g., “Where does the Miami-Dade Parks, Recreation, and Open Spaces Department move excess seaweed?”), and provided their impression of the article (e.g., “How interesting did you find this article?” 1 = *not at all*, 7 = *very interesting*). Participants also indicated how familiar they were with the news source, the *Quad City Times* (1 = *not at all*, 7 = *extremely*).

##### Dependent measures

Next, participants rated how politically biased they believed the article to be (1 = *not at all biased*, 7 = *extremely biased*), and in which direction they believed the bias to be (1 = *very conservatively biased*, 7 = *very liberally biased*). Then, participants rated how credible they believed the article and source to be (1 = *not at all credible*, 7 = *very credible*; credibility responses were aggregated to form a composite, *a* = 0.81). Participants also indicated how accurate they thought the bias indicator was (1 = *not at all accurate*, 7 = *extremely accurate*). Participants then responded to a number of exploratory questions about the bias indicators that are not reported here.

Finally, participants provided demographic data including their political identity and were debriefed.

## Results

In order to conduct our primary analyses, we collapsed across news article conditions^3^ and conducted a series of 2 (participant political ideology: liberal, conservative) × 4 (bias indicator: control, left, center, right) ANOVAs. See Table 1 for means and SDs of all dependent variables across experimental conditions and political ideology.

**TABLE 1:** Study 1 means and standard deviations across bias indicator conditions and participant political ideology.

	Control		Center		Right		Left	
**Dependent measures**	**Liberals**	**Conservatives**	**Liberals**	**Conservatives**	**Liberals**	**Conservatives**	**Liberals**	**Conservatives**
Credibility	5.24 (1.04)	4.94 (1.17)	5.16 (1.28)	5.22 (1.05)	4.64 (1.52)	5.36 (0.86)	4.75 (1.39)	5.06 (1.24)
Bias	2.66 (1.83)	2.33 (2.00)	2.54 (1.93)	3.20 (2.10)	2.70 (1.92)	3.50 (2.25)	2.98 (1.74)	3.51 (2.20)
Bias direction	4.30 (0.95)	4.32 (1.01)	4.22 (0.91)	4.45 (1.07)	4.04 (1.19)	4.50 (1.13)	4.52 (0.94)	4.51 (1.25)
Indicator accuracy	–	–	5.12 (1.57)	5.23 (1.66)	4.20 (1.68)	4.40 (1.71)	4.23 (1.46)	4.54 (1.85)

Standard deviations are in parentheses. All ratings made on 1–7 scale.

### RQ1: Do bias indicators influence how politically biased people perceive articles to be?

No, there was no effect of the bias indicator condition, *F* < 1, on participants’ perceptions of the bias of the article, nor was there a bias indicator x participant political ideology interaction, *F* < 1. Additionally, there was no significant effect of bias indicators on the perceived direction of bias, *F* < 1. There was not a significant effect of participant politics, *F*(1,341) = 2.26, *p* = 0.134, η_*p*_^2^ = 0.007; nor was there a significant politics x bias interaction, *F* < 1. While this finding could suggest that our bias indicator manipulation may not have been effective, it also could point to the fact that the articles we chose were, indeed, not-biased, and that the readers were picking up on that even with the bias indicators.

### RQ2: Do bias indicators influence how credible participants perceived the articles to be?

It depends on political identity. There was a political ideology × bias indicator interaction on participants’ credibility ratings, *F*(3,344) = 4.09, *p* = 0.042, η_*p*_^2^ = 0.023. Simple effects tests suggest that for conservatives, there was not a significant main effect of bias indicator condition, *F*(3,344) = 1.19, *p* = 0.316, η_*p*_^2^ = 0.022. For liberals, however, there was a marginal effect of the bias indicator, *F*(3,344) = 2.45, *p* = 0.065, η_*p*_^2^ = 0.037. *Post hoc* comparisons suggest that, in line with the world-view confirming hypothesis, articles with right-leaning indicators were viewed as less credible than the control (*p* = 0.026), and marginally less credible than those with left-leaning indicators (*p* = 0.056) or center-leaning indicators (*p* = 0.069). Neither the main effect of participants’ political ideology *F*(1,344) = 2.37, *p* = 0.124, η_*p*_^2^ = 0.007, nor of the bias indicator, *F*(3,344) = 0.86, *p* = 0.460, η_*p*_^2^ = 0.007 were significant. Overall, people found the articles to be credible, *M* = 4.97, *SD* = 1.24; a one-sample *t*-test revealed that participants rated the articles to be more credible than the midpoint of the scale (4), *t*(393) = 15.58, *p* < 0.001, *d* = 1.24.

### RQ3: Do people believe that the bias indicator is an accurate representation of the article bias?

Maybe. There was a main effect of the bias indicator condition on how accurate the participants perceived the bias indicator to be, *F*(1,249) = 7.08, *p* = 0.001, η_*p*_^2^ = 0.054. Participants in the center-leaning condition (*M* = 5.12, *SD* = 1.57) thought the indicator was more accurate than those in the right-leaning condition (*M* = 4.29, *SD* = 1.69, *p* = 0.001) and the left-leaning condition (*M* = 4.35, *SD* = 1.63, *p* = 0.001). As the articles we selected were all considered “center-leaning” (or unbiased), this result suggests that the participants were more likely to believe the bias indicator was accurate when it actually was presenting factually correct information. There was no significant effect of participant political ideology, *F*(1,249) = 1.00, *p* = 0.318, η_*p*_^2^ = 0.004, or interaction, *F* < 1, on perceived accuracy of the bias indicator.

## Discussion

Overall, Study 1 did not provide much evidence that bias indicators actually influenced the way that people read or perceive news articles. The one exception is that there was evidence in support of the worldview-confirming hypothesis, but unexpectedly only for liberal participants; they reported that articles labeled as biased (especially right-leaning) were less credible. We also saw evidence that conservative participants believed articles to have more political bias regardless of condition, consistent with the national trends that Republicans have higher distrust of the media, overall (Pew Research Center, 2020). The most notable effects found in Study 1, however, were that people seemed to feel that bias indicators themselves were less accurate when they suggested the articles were politically biased. Because the articles we chose were, in fact, center-leaning according to the bias indicators, our participants correctly identified the “center-leaning” bias indicator as being more accurate than the others. This suggests that people might not actually need a bias indicator to know when news is unbiased; however, because all articles were actually center-leaning, we do not know whether bias indicators would be important for interpreting actually left or right-leaning articles. Additionally, because our study was underpowered, it is possible that bias indicators do have a small effect on perceptions of news that we were not able to capture.

## Study 2

### Overview

The goal of Study 2 was to test how bias indicators influence the way people read and perceive news articles that actually contain political bias. Study 1 used unbiased articles, and participants accurately did not perceive the articles labeled as biased to contain political bias (more than the center-leaning or control), nor did they perceive the bias indicators to be accurate if they signified bias. As such, it is important to test whether bias indicators are more persuasive or believable to readers when the articles actually contain a political slant. In Study 2, participants read one of two articles on the same partisan topic that were either right or left-leaning, and were told that the articles were either biased (correct information) or not (incorrect information) before rating the credibility of the article, and their perceptions of the indicators themselves. A secondary goal of Study 2 was to examine how people believe they would use bias indicators in the future. Finally, Study 2 improved upon Study 1 by increasing statistical power and improving sample size.

### Materials and methods

#### Participants and design

U.S. American MTurk workers (*N* = 580) were recruited via Cloud Research and compensated $2.50. We specifically recruited participants who were pre-screened as Democrats or Republicans in an attempt to capture people whose ideologies leaned left or right, and to avoid losing as many participants as we did in Study 1. An *a priori* power analysis (Faul et al., 2007) assuming a small effect size (as determined by Study 1), determined that a total sample size of *N* = 432 was needed to achieve 80% power. Because in Study 1 our sample leaned liberal and we lost several participants for not having a political leaning, we recruited additional participants to compensate. Participants ranged in ages from 19 to 78 (*M*_age_ = 38.44 years, *SD*_age_ = 12.08 years), and were majority male (63.9%) and white (75.00%; 16.20% were Black or African-American, 9.64% were Hispanic or Latino, 5.68% were Asian, and less than 2% were other races). Participants’ areas of residence largely followed typical demographic trends in the USA with most participants living in the South (25.9%), the West (17.8%) followed by the Midwest (16.6%), East Coast (15.4%), and the Mountain region (4.6%). Inconsistent with the nationwide demographics (Pew Research Center, 2020), more participants identified as Republican (43.8%) than Democrat (36.7%). Despite specifically recruiting participants who had previously self-identified as Democrats or Republicans, a significant portion of our sample identified as Independent or Other (19.5%). As in Study 1, we bifurcated the political ideology of participants in order to include those who identified as Independent (*N* = 263 liberals, *N* = 270 conservatives) and dropped participants who identified as neither (*N* = 83), leaving a total sample of 533.

Most participants (64.3%) reported not having previous experience with bias indicators. Upon starting the study, participants were randomly assigned to one of six conditions in a 2 (Actual Bias: left, right) × 3 (Bias Indicator: control, center, biased) between-subjects design.

#### Materials

##### Articles

In Study 2, the research team selected articles on partisan topics; each was about 600 words long. The research team found one left-leaning and one right-leaning article (according to the NoBias chrome extension) on each of three political topics: The visit of Kent State’s “Gun Girl” to Ohio University (Rahman, 2020; Wallace, 2020), the Arizona supreme court case regarding businesses refusing to serve same-sex couples (NBC Universal News Group, 2019; Parke, 2019), and the Louisiana “heartbeat” bill struck down by the state supreme court (Berry, 2019; Borter and Dobuzinskis, 2019). As in Study 1, news articles were re-formatted to reflect the *Quad City Times*, a center-leaning, small, Midwestern regional news source, to standardize the effects of the source of the articles.

##### Bias indicators

The bias indicator manipulation was included in the margins at 3 points on each of the articles for participants not in the control condition. The bias indicators either indicated that the article was center-leaning, or that the article leaned left or right (whatever the true lean of the article was). In other words, participants in the bias condition saw the *actual* bias associated with the article.

### Procedure

The procedure was largely the same as in Study 1. First, participants read their assigned article and answered 4 multiple choice questions as part of the cover story testing their comprehension of the articles. These questions were the same across both the left and right versions of each article; and were broad enough that they could be answered by both articles. Next, participants responded to the same questions about the political bias and credibility of the articles and source as in Study 1 (1 = *not at all*, 7 = *extremely*; *a* = 0.88).

#### Perceptions of bias indicators

Participants who received bias indicators also rated how accurate they thought the bias indicator was, and whether the indicators made the article seem more credible (1 = *not at all*, 7 = *extremely*).

As in Study 1, participants also responded to several exploratory questions about bias indicators that are not discussed here.

#### Behavioral intentions

Participants also rated their likelihood of choosing a center, right, or left-leaning article if they were to use a bias indicator in the future (1 = *not at all*, 7 = *extremely*).

## Results

To conduct our primary analyses, we collapsed across news article topic conditions^4^ and conducted a series of 2 (participant’s political ideology: liberal, conservative) × 2 (actual bias: left, right) × 3 (bias indicator: control, center, biased) ANOVAs. See Table 2 for means and SDs of all variables across experimental conditions and political ideology.

**TABLE 2:** Study 2 means and standard deviations of dependent measures across bias indicator and political conditions.

	Bias indicator	Biased		Center		Control (No indicator)	
**Dependent measures**	**Actual bias**	**Left**	**Right**	**Left**	**Right**	**Left**	**Right**
Credibility	Liberal participants	4.84 (1.23)	5.04 (1.17)	5.15 (1.02)	4.64 (1.15)	4.65 (1.72)	4.72 (1.48)
	Conservative participants	4.88 (1.27)	5.50 (1.09)	5.28 (1.29)	5.14 (1.23)	5.04 (1.09)	5.32 (1.90)
Bias amount	Liberal participants	4.11 (1.79)	4.47 (1.9)	3.84 (1.89)	4.06 (1.87)	3.70 (1.90)	4.32 (1.87)
	Conservative participants	4.20 (1.87)	4.49 (1.53)	4.55 (1.86)	4.33 (1.85)	4.49 (1.93)	4.52 (2.01)
Bias direction	Liberal participants	4.69 (1.31)	4.21 (1.73)	4.48 (1.23)	4.64 (1.43)	4.25 (1.50)	3.87 (1.68)
	Conservative participants	4.72 (1.31)	4.19 (1.80)	4.24 (1.60)	4.65 (1.55)	4.87 (1.38)	4.95 (1.41)
Indicator accuracy	Liberal participants	4.63 (1.70)	5.17 (1.28)	5.26 (1.43)	4.94 (1.50)	−	−
	Conservative participants	4.65 (1.74)	5.25 (1.40)	5.10 (1.40)	5.14 (1.58)	−	−

Standard deviations are in parentheses. All ratings made on 1–7 scale.

### RQ1: Do bias indicators influence how politically biased people perceive articles to be?

No. Only participant political ideology predicted perceptions of bias, *F*(1,521) = 4.50, *p* = 0.034, η_p_^2^ = 0.009. Consistent with national trends, conservatives (*M* = 4.41, *SD* = 1.82) perceived more bias in the articles than liberals (*M* = 4.06, *SD* = 1.87), overall, regardless of whether bias indicators were present.

However, there were effects of the bias indicator on which type of political bias people perceived. Specifically there was a bias indicator x participant politics interaction, *F*(2,509) = 5.12, *p* = 0.006, η_p_^2^ = 0.02. Liberal participants perceived the control articles (*M* = 4.05, *SD* = 1.60) to be more conservatively biased than those with a center-leaning label (*M* = 4.54, *SD* = 1.34; *p* = 0.029). Conservative participants perceived the control articles (*M* = 4.91, *SD* = 1.39) to be more liberally biased than those with biased labels (*M* = 4.45, *SD* = 1.59; *p* = 0.038).

### RQ2: Do bias indicators influence how credible participants viewed the articles?

Maybe. There was not a main effect of bias indicator on perceptions of credibility, *F* < 1; however, there was a significant bias indicator x actual bias interaction, *F*(2,521) = 4.15, *p* = 0.016, η_p_^2^ = 0.016. In line with the media literacy hypothesis, simple effects tests revealed that for participants who viewed left-leaning articles, there was a main effect of bias indicator, *F*(2,310) = 2.98, *p* = 0.052, η_p_^2^ = 0.019, such that they found the articles more credible when they had a center-leaning indicator (*M* = 5.19, *SD* = 1.11) than biased (*M* = 4.86, *SD* = 1.25; *p* = 0.046) or control (*M* = 4.84, *SD* = 1.14; *p* = 0.029). There was no effect of bias indicator for participants who viewed the right-leaning articles, *F*(2,303) = 1.68, *p* = 0.189, η_p_^2^ = 0.011. As in Study 1, there was also a main effect of participants’ political ideology on perceptions of credibility, *F*(1,521) = 11.20, *p* = 0.001, η_p_^2^ = 0.021. However, in this case, conservative participants perceived more credibility (*M* = 5.19, *SD* = 1.18) than liberals (*M* = 4.86, *SD* = 1.21). No other main effects or interactions were significant.

### RQ3: Do people believe that the bias indicator is an accurate representation of the article bias?

Maybe. Overall, participants who received bias indicators rated them as more accurate (*M* = 4.99, *SD* = 1.53) than the midpoint of the scale (4), *t*(414) = 66.49, *p* < 0.001, *d* = 1.53. However, the perceived accuracy depended on the actual bias of the article. That is, there was a bias indicator x actual bias interaction on perceptions of accuracy, *F*(1,355) = 4.67, *p* = 0.031, η_p_^2^ = 0.013. Simple effect tests indicated that, among participants who viewed the left-leaning articles, there was a main effect of bias indicator, *F*(1,213) = 9.21, *p* = 0.003, η_p_^2^ = 0.041, such that articles with the center-leaning indicator (*M* = 5.22, *SD* = 1.43) were viewed as more accurate than those with the left-leaning bias indicator (*M* = 4.56, *SD* = 1.72). For participants who viewed the right-leaning article, there was not an effect of bias indicator on perceptions of accuracy, *F* < 1.

### RQ4: How do people predict they would use bias indicators in the future?

**FIGURE 2:**
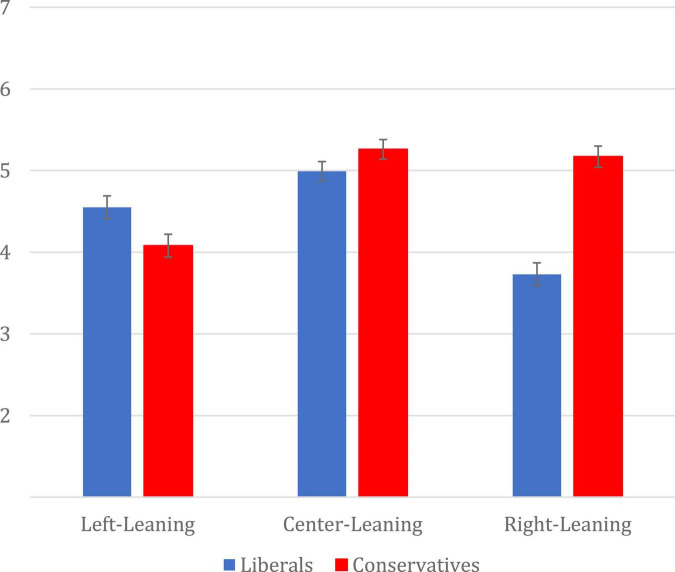
Mean likelihood ratings of choosing left, center, or right-leaning articles by political ideology. Error bars represent standard error.

In order to test what types of articles people predicted they would use a bias indicator to choose in the future, we conducted a 3 (Choice: left, center, right) × 2 (Participant Partisanship: liberal, conservative) Repeated Measures ANOVA where choice was within-subjects and politics was between-subjects. There was a main effect of choice, *F*(2,722) = 23.83, *p* < 0.001, η_p_^2^ = 0.062, such that participants reported a greater likelihood of choosing center-leaning articles (*M* = 5.13, *SD* = 1.62) than left-leaning articles (*M* = 4.31, *SD* = 1.91, *p* < 0.001) and right-leaning articles (*M* = 4.47, *SD* = 1.97, *p* < 0.001). However, a significant choice x politics interaction also emerged, *F*(2,722) = 29.21, *p* < 0.001, η_p_^2^ = 0.075; See Figure 2.

Simple effects tests reveal that for liberals, there was a main effect of Choice, *F*(2,350) = 24.31, *p* < 0.001, η_p_^2^ = 0.122. *Post hoc* comparisons reveal that liberals reported being more likely to choose a center-leaning article (*M* = 4.99, *SD* = 1.69) than a left-leaning (*M* = 4.55, *SD* = 1.69, *p* = 0.008), or right-leaning (*M* = 3.73, *SD* = 2.03, *p* < 0.001). However, they were also more likely to report wanting to choose a left-leaning article over a right-leaning article (*p* < 0.001). For conservatives there was also a main effect of Choice, *F*(2,372) = 28.92, *p* < 0.001, η_p_^2^ = 0.135, such that they reported being more likely to choose a center-leaning (*M* = 5.26, *SD* = 1.55) or right-leaning (*M* = 5.17, *SD* = 1.64) than a left-leaning (*M* = 4.08, *SD* = 2.08, *p*s < 0.001). These findings are consistent with the world-view confirming hypothesis; both liberals and conservatives reported they would be more likely to use a bias indicator to choose an article that leaned in their political direction than one that opposed.

## Discussion

Study 2 provided more evidence that bias indicators do not generally influence the way that people perceive news articles. One exception is that left-leaning articles were considered to be more credible when they were described as being “center-leaning” versus left-biased or not including a bias indicator. This finding is consistent with the media-literacy hypothesis that describing something as biased makes it seem less credible.

Like in Study 1, we see some evidence that people didn’t believe the bias indicator; it was considered most believable for people in the “center-leaning” condition. Because the articles chosen for this study actually did contain bias, this was unexpected.

Notably, we also saw evidence that participants thought they would use bias indicators in the future in a worldview-confirming way. That is, people reported that they would be less likely to use bias indicators in the future to seek out counter-attitudinal news (and “balance” their media diet, as bias indicator sites suggest), and would instead be more likely to choose center-leaning or pro-attitudinal news pieces.

### General discussion

Across two experiments, we tested whether bias indicators influence readers’ perceptions of news. Study 1, tested how bias indicators affect perceptions of center-leaning articles, while Study 2 tested how bias indicators affect perceptions of actually biased articles. Across both studies, we saw little to no evidence that bias indicators actually change how people read or perceive articles, though they do affect other associated behavioral factors.

We saw some slight evidence that liberals view conservative-labeled (i.e., right-leaning) pieces as less credible, consistent with a worldview-confirming hypothesis, but we also saw some slight evidence that people view liberal-leaning articles as more credible when labeled as leaning center, consistent with the framing hypothesis. Neither result was consistent across both studies, suggesting that actual political bias (or lack thereof) may be more influential to readers’ perceptions than bias indicators.

Notably, in Study 2, people reported that they would be more likely to use bias indicators in the future to choose center-leaning or pro-attitudinal articles, consistent with the worldview-confirming hypothesis. While this finding did not suggest that bias indicators actually influence how people read or perceive news, it does suggest that it might influence how people *choose* the news they read, and not how bias indicator creators assume. While most bias indicator companies develop their products to reduce media bias or help people have a more balanced “media-diet,” this data suggests that instead, U.S. Americans might be motivated to use bias indicators in order to become *more* biased and increase their selective partisan exposure (e.g., Arendt et al., 2019). Future research should examine how bias indicators influence article choices in this way. Allowing participants to freely select which articles to read would increase ecological validity by more closely mirroring the functionality of bias indicators in the real world.

## Limitations

Our work is not without limitations. First is our convenient MTurk sample; there has been a significant decrease in quality participants from MTurk in recent years (see Chmielewski and Kucker, 2020), namely, in that they do not pay close attention to the studies they are participating in. Future research would ideally be performed in a lab setting to offer more control. While doing so would encourage a more focused environment, it is important to acknowledge that real news consumers also may not be highly focused while scrolling through headlines and viewing the indicators.

Additionally, the articles that were chosen in both studies were *real* articles, which means that we sacrificed experimental control over what the participants read in favor of more external validity. While we relied on the NoBias algorithm to determine the political slant of the articles, future research should pre-test articles with participants to ensure they are perceived as neutral or biased.

Additionally, while the articles we chose had topics still relevant to the time period we ran participants in, they were not necessarily as recent as news would be on an active news media site, and we did not capture measures of personal involvement with or familiarity with the different topics. Also, we purposefully chose articles that did not have broad national coverage with hopes that our participants did not have pre-existing attitudes about the stories. As a result of both of these factors, participants may not have been as engaged in the articles chosen as if they were breaking news or more nationally relevant. Future research should test whether and how these factors influence the use and effects of bias indicators.

Another limitation of our work was that we only included liberals and conservatives in our sample, and not moderates. Our primary interest was on how U.S. partisans respond to bias indicators that either matched their political perspective or not, in line with the goals of many bias indicator apps and sites. However, including moderates in future studies could provide insights about the types of media they typically consume and whether they use bias indicators differently than partisans. Presumably, moderates would be more motivated to focus on center-leaning news, or to expose themselves to a balance of left- and right-leaning sources.

## Conclusion

Political bias indicators for news sites and articles are being designed as a response situated in the U.S. American news literacy movement. However, the present studies provide initial evidence that political bias indicators might not be having the intended effects. The present data suggests that, at best, bias indicators do not actually influence people’s perceptions of news media, and at worst, they might actually increase people’s exposure to biased news sources. This preliminary evidence suggests that political bias indicator sites or extensions may not predictably drive users to become less biased in their consumption of news; in fact, they may be counterproductive, which would become an ethical concern for bias indicator design. Organizations that make bias indicators may opt to provide more transparency about not only the purpose of these products, but the observed effects. For example, showing users data about how they used the bias indicator tools (e.g., did they read more articles that leaned left, right, or center) might help people realize their biased behaviors. While more work needs to be done to understand these effects, initiatives interested in creating bias indicators or other interventions that signal media-bias should take these studies into account when designing and implementing their tools.

## Data availability statement

The raw data supporting the conclusions of this article will be made available by the authors, without undue reservation.

## Ethics statement

The studies involving human participants were reviewed and approved by the Santa Clara University IRB. The patients/participants provided their written informed consent to participate in this study.

## Author contributions

KB organized the data. KB and AF performed the statistical analysis and wrote the first draft of the manuscript. All authors contributed to conception and design of the study, manuscript revision, read, and approved the submitted version.

## Footnotes

1 We will use the terms “liberal” and “conservative,” as well as “political ideology” and “political beliefs” throughout this manuscript when describing our own study and hypotheses; but might describe “Democrats” and “Republicans” when describing other research or our participants’ self-identified group membership. We recognize that examining political ideology instead of identity or partisanship might lead to more variability in the data (as “liberals” and “conservatives” are not monolithic groups with a single set of beliefs or identities), but political bias indicators examine an ideological slant in news more so than categorizing news as being “Republican” or “Democrat.”

2 The source credibility was held constant across articles in order to explicitly test how bias indicators of the articles themselves influenced perceptions of the articles.

3 While the articles were rated slightly differently on items like interest, the article variable did not interact with our main variables of interest.

4 As in Study 1, the news topic conditions did not interact with any of our variables of interest.
